# Di-μ-nicotinamide-κ^2^ *O*:*N* ^1^;κ^2^ *N* ^1^:*O*-bis­[aqua­bis­(4-meth­oxy­benzoato-κ*O*)copper(II)]

**DOI:** 10.1107/S1600536810022415

**Published:** 2010-06-16

**Authors:** Tuncer Hökelek, Yasemin Süzen, Barış Tercan, Erdinç Tenlik, Hacali Necefoğlu

**Affiliations:** a Department of Physics, Hacettepe University, 06800 Beytepe, Ankara, Turkey; b Department of Chemistry, Faculty of Science, Anadolu University, 26470 Yenibağlar, Eskişehir, Turkey; c Department of Physics, Karabük University, 78050 Karabük, Turkey; d Department of Chemistry, Kafkas University, 63100 Kars, Turkey

## Abstract

The asymmetric unit of the centrosymmetric dinuclear title compound, [Cu_2_(C_8_H_7_O_3_)_4_(C_6_H_6_N_2_O)_2_(H_2_O)_2_], contains one half of the complex mol­ecule. Each Cu^II^ atom is five-coordinated by one N atom from one bridging nicotinamide ligand and one O atom from another symmetry-related bridging nicotinamide ligand, two O atoms from two 4-meth­oxy­benzoate ligands, and one water mol­ecule, forming a distorted square-pyramidal geometry. Inter­molecular O—H⋯O and N—H⋯O hydrogen bonds link the mol­ecules into layers parallel to (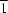
01). π–π inter­actions, indicated by short inter­molecular distances of 3.801 (1) Å between the centroids of the benzene rings and 3.653 (1) Å between the centroids of the pyridine rings, further stabilize the structure.

## Related literature

For related structures, see: Hökelek & Necefoğlu (1996 ▶); Hökelek *et al.* (2009*a* ▶,*b* ▶,*c* ▶,*d* ▶).
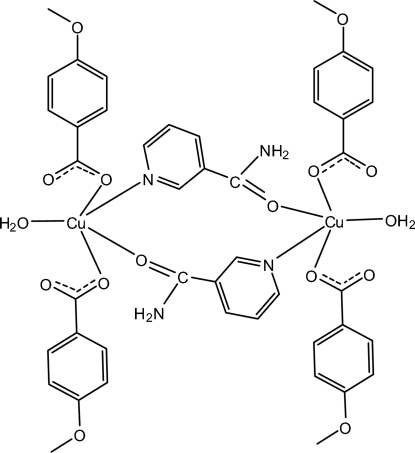


## Experimental

### 

#### Crystal data

[Cu_2_(C_8_H_7_O_3_)_4_(C_6_H_6_N_2_O)_2_(H_2_O)_2_]
*M* *_r_* = 1011.93
Monoclinic, 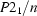

*a* = 14.1707 (3) Å
*b* = 8.4319 (2) Å
*c* = 18.0225 (3) Å
β = 95.847 (2)°
*V* = 2142.23 (8) Å^3^
*Z* = 2
Mo *K*α radiation
μ = 1.07 mm^−1^
*T* = 100 K
0.37 × 0.37 × 0.23 mm

#### Data collection

Bruker Kappa APEXII CCD area-detector diffractometer
Absorption correction: multi-scan (*SADABS*; Bruker, 2005 ▶) *T*_min_ = 0.678, *T*_max_ = 0.781
20329 measured reflections
5403 independent reflections
4813 reflections with *I* > 2σ(*I*)
*R*_int_ = 0.021

#### Refinement

*R*[*F* ^2^ > 2σ(*F* ^2^)] = 0.026
*wR*(*F* ^2^) = 0.072
*S* = 1.05
5403 reflections
316 parameters
H atoms treated by a mixture of independent and constrained refinement
Δρ_max_ = 0.47 e Å^−3^
Δρ_min_ = −0.29 e Å^−3^

### 

Data collection: *APEX2* (Bruker, 2007 ▶); cell refinement: *SAINT* (Bruker, 2007 ▶); data reduction: *SAINT*; program(s) used to solve structure: *SHELXS97* (Sheldrick, 2008 ▶); program(s) used to refine structure: *SHELXL97* (Sheldrick, 2008 ▶); molecular graphics: *Mercury* (Macrae *et al.*, 2006 ▶); software used to prepare material for publication: *WinGX* (Farrugia, 1999 ▶) and *PLATON* (Spek, 2009 ▶).

## Supplementary Material

Crystal structure: contains datablocks I, global. DOI: 10.1107/S1600536810022415/cv2731sup1.cif

Structure factors: contains datablocks I. DOI: 10.1107/S1600536810022415/cv2731Isup2.hkl

Additional supplementary materials:  crystallographic information; 3D view; checkCIF report

## Figures and Tables

**Table 1:** Hydrogen-bond geometry (Å, °)

*D*—H⋯*A*	*D*—H	H⋯*A*	*D*⋯*A*	*D*—H⋯*A*
N2—H2*A*⋯O1^i^	0.86 (2)	2.03 (2)	2.8407 (18)	158 (2)
N2—H2*B*⋯O4^ii^	0.83 (2)	2.29 (2)	2.9897 (17)	141.4 (18)
O8—H81⋯O1^iii^	0.79 (3)	1.97 (3)	2.7236 (15)	159 (3)
O8—H82⋯O4^iii^	0.825 (18)	1.803 (18)	2.6052 (16)	163.9 (18)

Symmetry codes: (i) 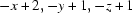; (ii) 
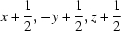
; (iii) 
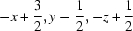
.

**Fig. 1.**
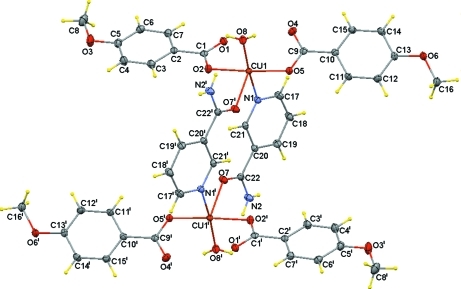
The molecular structure of the title molecule with the atom-numbering scheme. Displacement ellipsoids are drawn at the 50% probability level [symmetry code: (') 2 - x, -y, 1 - z].

## supplementary crystallographic information

### Comment

As a part of our ongoing study of transition metal complexes of nicotinamide (Hökelek & Necefoğlu, 1996; Hökelek *et al.*, 2009*a, b, c, d*), herein we report the crystal structure of the title dinuclear complex.

The title compound, (I), consists of dimeric units located around a crystallographic symmetry centre and made up of two Cu cations, four 4-methoxybenzoate (MB) anions, two nicotinamide (NA) ligands and two water molecules (Fig. 1). Both of the Cu^II^ centres are five-coordinated with distorted square-pyramidal environments, and the two monomeric units are bridged through the two nicotinamide (NA) ligands about an inversion center. The Cu1···Cu1^i^ (symmetry code: (i) 2 - *x*, -*y*, 1 - *z*) distance is 7.1368 (3) Å. The average Cu—O bond length is 2.0626 (10) Å, and the Cu atom is displaced out of the least-squares planes of the carboxylate groups (O1/C1/O2) and (O4/C9/O5) by 0.0015 (2) and -0.2589 (2) Å, respectively.

The dihedral angles between the planar carboxylate groups and the adjacent benzene rings A (C2—C7) and B (C10—C15) are 1.85 (5) and 10.16 (7) °, respectively, while those between rings A, B and C (N1/C17—C21) are A/B = 28.50 (4), A/C = 81.64 (4), B/C = 58.50 (4) °.

In the crystal structure, intermolecular O—H···O and N—H···O hydrogen bonds (Table 1) link the molecules into layers. The π–π contacts between the benzene rings and between the pyridine rings, *Cg*2—*Cg*2^i^ and *Cg*3—*Cg*3^ii^ [symmetry codes: (i) 2 - *x*, 2 - *y*, -*z*; (ii) 1 - *x*, 2 - *y*, -*z*, where *Cg*2 and *Cg*3 are the centroids of the rings B (C10—C15) and C (N1/C17—C21)] may further stabilize the structure, with centroid-centroid distances of 3.801 (1) and 3.653 (1) Å, respectively.

### Experimental

The title compound was prepared by the reaction of CuSO_4_.5H_2_O (2.50 g, 10 mmol) in H_2_O (50 ml) and NA (2.44 g, 20 mmol) in H_2_O (50 ml) with sodium 4-methoxybenzoate (3.48 g, 20 mmol) in H_2_O (100 ml). The mixture was filtered and set aside to crystallize at ambient temperature for one week, giving blue single crystals.

### Refinement

Atoms H81, H82 (for H_2_O) and H2A, H2B (for NH_2_) were located in difference Fourier maps and refined isotropically. The remaining H atoms were positioned geometrically with C—H = 0.93 and 0.96 Å for aromatic and methyl H atoms, respectively, and constrained to ride on their parent atoms, with *U*_iso_(H) = *xU*_eq_(C), where *x* = 1.5 for methyl H and *x* = 1.2 for aromatic H atoms.

### Crystal data

**Table:**

[Cu_2_(C_8_H_7_O_3_)_4_(C_6_H_6_N_2_O)_2_(H_2_O)_2_]	*F*(000) = 1044
*M**_r_* = 1011.93	*D*_x_ = 1.569 Mg m^−^^3^
Monoclinic, *P*2_1_/*n*	Mo *K*α radiation, λ = 0.71073 Å
Hall symbol: -P 2yn	Cell parameters from 9977 reflections
*a* = 14.1707 (3) Å	θ = 2.7–28.5°
*b* = 8.4319 (2) Å	µ = 1.07 mm^−^^1^
*c* = 18.0225 (3) Å	*T* = 100 K
β = 95.847 (2)°	Block, blue
*V* = 2142.23 (8) Å^3^	0.37 × 0.37 × 0.23 mm
*Z* = 2	

### Data collection

**Table:**

Bruker Kappa APEXII CCD area-detector diffractometer	5403 independent reflections
Radiation source: fine-focus sealed tube	4813 reflections with *I* > 2σ(*I*)
graphite	*R*_int_ = 0.021
φ and ω scans	θ_max_ = 28.5°, θ_min_ = 1.7°
Absorption correction: multi-scan (*SADABS*; Bruker, 2005)	*h* = −18→18
*T*_min_ = 0.678, *T*_max_ = 0.781	*k* = −10→11
20329 measured reflections	*l* = −24→24

### Refinement

**Table:**

Refinement on *F*^2^	Primary atom site location: structure-invariant direct methods
Least-squares matrix: full	Secondary atom site location: difference Fourier map
*R*[*F*^2^ > 2σ(*F*^2^)] = 0.026	Hydrogen site location: inferred from neighbouring sites
*wR*(*F*^2^) = 0.072	H atoms treated by a mixture of independent and constrained refinement
*S* = 1.05	*w* = 1/[σ^2^(*F*_o_^2^) + (0.0384*P*)^2^ + 1.1134*P*] where *P* = (*F*_o_^2^ + 2*F*_c_^2^)/3
5403 reflections	(Δ/σ)_max_ = 0.001
316 parameters	Δρ_max_ = 0.47 e Å^−^^3^
0 restraints	Δρ_min_ = −0.29 e Å^−^^3^

### Special details

**Table:**

Geometry. All e.s.d.'s (except the e.s.d. in the dihedral angle between two l.s. planes) are estimated using the full covariance matrix. The cell e.s.d.'s are taken into account individually in the estimation of e.s.d.'s in distances, angles and torsion angles; correlations between e.s.d.'s in cell parameters are only used when they are defined by crystal symmetry. An approximate (isotropic) treatment of cell e.s.d.'s is used for estimating e.s.d.'s involving l.s. planes.
Refinement. Refinement of *F*^2^ against ALL reflections. The weighted *R*-factor *wR* and goodness of fit *S* are based on *F*^2^, conventional *R*-factors *R* are based on *F*, with *F* set to zero for negative *F*^2^. The threshold expression of *F*^2^ > σ(*F*^2^) is used only for calculating *R*-factors(gt) *etc*. and is not relevant to the choice of reflections for refinement. *R*-factors based on *F*^2^ are statistically about twice as large as those based on *F*, and *R*- factors based on ALL data will be even larger.

### Fractional atomic coordinates and isotropic or equivalent isotropic displacement parameters (Å^2^)

**Table:**

	*x*	*y*	*z*	*U*_iso_*/*U*_eq_	
Cu1	0.832076 (11)	0.152692 (19)	0.356751 (8)	0.00986 (6)	
O1	0.89942 (7)	0.40998 (13)	0.27780 (5)	0.0155 (2)	
O2	0.94951 (7)	0.16598 (12)	0.30792 (5)	0.01368 (19)	
O3	1.28693 (8)	0.39532 (15)	0.12796 (7)	0.0251 (2)	
O4	0.66614 (8)	0.34649 (13)	0.32513 (6)	0.0181 (2)	
O5	0.71138 (7)	0.14541 (12)	0.40038 (5)	0.01317 (19)	
O6	0.32798 (7)	0.34252 (13)	0.52920 (6)	0.0170 (2)	
O7	1.10015 (7)	0.07805 (12)	0.58526 (5)	0.0153 (2)	
O8	0.77027 (8)	0.04960 (14)	0.26669 (6)	0.0167 (2)	
H81	0.7200 (18)	0.008 (3)	0.2652 (13)	0.047 (7)*	
H82	0.7977 (13)	−0.001 (2)	0.2360 (10)	0.017 (4)*	
N1	0.88593 (8)	0.27822 (14)	0.44653 (6)	0.0118 (2)	
N2	1.09767 (9)	0.27022 (17)	0.67244 (7)	0.0171 (3)	
H2A	1.0823 (15)	0.365 (3)	0.6840 (12)	0.029 (5)*	
H2B	1.1375 (14)	0.224 (2)	0.7019 (11)	0.022 (5)*	
C1	0.95896 (9)	0.30016 (17)	0.27686 (7)	0.0125 (3)	
C2	1.04648 (9)	0.32640 (17)	0.23831 (7)	0.0125 (3)	
C3	1.11660 (10)	0.20966 (18)	0.23693 (8)	0.0152 (3)	
H3	1.1096	0.1132	0.2608	0.018*	
C4	1.19625 (10)	0.23685 (19)	0.20030 (8)	0.0187 (3)	
H4	1.2429	0.1594	0.2003	0.022*	
C5	1.20666 (10)	0.38071 (19)	0.16333 (8)	0.0173 (3)	
C6	1.13758 (10)	0.49779 (18)	0.16412 (8)	0.0166 (3)	
H6	1.1442	0.5936	0.1396	0.020*	
C7	1.05849 (10)	0.46977 (18)	0.20197 (7)	0.0153 (3)	
H7	1.0126	0.5484	0.2031	0.018*	
C8	1.29541 (12)	0.5321 (2)	0.08255 (9)	0.0254 (3)	
H8A	1.3544	0.5279	0.0607	0.038*	
H8B	1.2938	0.6258	0.1126	0.038*	
H8C	1.2437	0.5346	0.0437	0.038*	
C9	0.65288 (9)	0.25480 (17)	0.37733 (7)	0.0123 (3)	
C10	0.56660 (9)	0.27358 (16)	0.41748 (7)	0.0121 (2)	
C11	0.55839 (10)	0.19292 (18)	0.48349 (8)	0.0148 (3)	
H11	0.6064	0.1240	0.5019	0.018*	
C12	0.47980 (10)	0.21312 (18)	0.52268 (8)	0.0159 (3)	
H12	0.4753	0.1586	0.5670	0.019*	
C13	0.40798 (10)	0.31546 (17)	0.49503 (8)	0.0134 (3)	
C14	0.41505 (10)	0.39815 (18)	0.42856 (8)	0.0156 (3)	
H14	0.3669	0.4669	0.4102	0.019*	
C15	0.49373 (10)	0.37730 (17)	0.39027 (8)	0.0142 (3)	
H15	0.4985	0.4324	0.3461	0.017*	
C16	0.32415 (10)	0.26991 (19)	0.60087 (8)	0.0190 (3)	
H16A	0.2661	0.2992	0.6205	0.028*	
H16B	0.3771	0.3052	0.6343	0.028*	
H16C	0.3267	0.1567	0.5958	0.028*	
C17	0.83872 (10)	0.40389 (17)	0.47003 (8)	0.0152 (3)	
H17	0.7874	0.4437	0.4394	0.018*	
C18	0.86367 (10)	0.47614 (18)	0.53812 (8)	0.0169 (3)	
H18	0.8306	0.5645	0.5524	0.020*	
C19	0.93883 (10)	0.41469 (17)	0.58490 (8)	0.0153 (3)	
H19	0.9555	0.4591	0.6316	0.018*	
C20	0.98863 (9)	0.28598 (16)	0.56074 (7)	0.0114 (2)	
C21	0.96065 (9)	0.22322 (16)	0.49060 (7)	0.0117 (2)	
H21	0.9953	0.1395	0.4735	0.014*	
C22	1.06782 (9)	0.20324 (17)	0.60760 (7)	0.0120 (2)	

### Atomic displacement parameters (Å^2^)

**Table:**

	*U*^11^	*U*^22^	*U*^33^	*U*^12^	*U*^13^	*U*^23^
Cu1	0.00880 (8)	0.01102 (9)	0.00963 (8)	0.00027 (6)	0.00037 (6)	−0.00144 (6)
O1	0.0127 (4)	0.0164 (5)	0.0175 (5)	0.0029 (4)	0.0014 (4)	−0.0019 (4)
O2	0.0120 (4)	0.0141 (5)	0.0153 (4)	−0.0002 (4)	0.0033 (4)	−0.0005 (4)
O3	0.0202 (5)	0.0259 (6)	0.0315 (6)	−0.0008 (5)	0.0141 (5)	0.0034 (5)
O4	0.0195 (5)	0.0198 (6)	0.0158 (5)	−0.0003 (4)	0.0055 (4)	0.0042 (4)
O5	0.0114 (4)	0.0150 (5)	0.0133 (4)	0.0011 (4)	0.0024 (3)	−0.0006 (4)
O6	0.0133 (5)	0.0200 (5)	0.0183 (5)	0.0049 (4)	0.0049 (4)	0.0030 (4)
O7	0.0165 (5)	0.0120 (5)	0.0168 (5)	0.0028 (4)	−0.0017 (4)	−0.0015 (4)
O8	0.0116 (5)	0.0229 (6)	0.0154 (5)	−0.0010 (4)	0.0013 (4)	−0.0084 (4)
N1	0.0112 (5)	0.0113 (6)	0.0128 (5)	0.0002 (4)	0.0002 (4)	−0.0008 (4)
N2	0.0184 (6)	0.0162 (7)	0.0155 (6)	0.0042 (5)	−0.0048 (5)	−0.0034 (5)
C1	0.0118 (6)	0.0150 (6)	0.0102 (5)	−0.0009 (5)	−0.0017 (5)	−0.0029 (5)
C2	0.0113 (6)	0.0147 (7)	0.0113 (6)	−0.0004 (5)	0.0001 (5)	−0.0018 (5)
C3	0.0154 (6)	0.0133 (7)	0.0171 (6)	0.0007 (5)	0.0025 (5)	0.0006 (5)
C4	0.0154 (6)	0.0186 (7)	0.0224 (7)	0.0039 (6)	0.0042 (5)	0.0005 (6)
C5	0.0139 (6)	0.0213 (7)	0.0172 (6)	−0.0031 (6)	0.0046 (5)	−0.0014 (6)
C6	0.0191 (7)	0.0153 (7)	0.0155 (6)	−0.0014 (5)	0.0025 (5)	0.0012 (5)
C7	0.0156 (6)	0.0156 (7)	0.0147 (6)	0.0013 (5)	0.0010 (5)	−0.0008 (5)
C8	0.0256 (8)	0.0279 (9)	0.0243 (7)	−0.0082 (7)	0.0101 (6)	0.0011 (7)
C9	0.0124 (6)	0.0130 (6)	0.0112 (6)	−0.0021 (5)	0.0001 (5)	−0.0027 (5)
C10	0.0117 (6)	0.0118 (6)	0.0128 (6)	−0.0004 (5)	0.0009 (5)	−0.0007 (5)
C11	0.0139 (6)	0.0156 (7)	0.0149 (6)	0.0048 (5)	0.0021 (5)	0.0026 (5)
C12	0.0175 (7)	0.0165 (7)	0.0141 (6)	0.0036 (5)	0.0037 (5)	0.0038 (5)
C13	0.0117 (6)	0.0136 (7)	0.0153 (6)	0.0005 (5)	0.0028 (5)	−0.0015 (5)
C14	0.0140 (6)	0.0141 (7)	0.0184 (6)	0.0033 (5)	−0.0005 (5)	0.0024 (5)
C15	0.0149 (6)	0.0137 (7)	0.0138 (6)	0.0005 (5)	0.0002 (5)	0.0031 (5)
C16	0.0178 (7)	0.0209 (8)	0.0194 (7)	0.0025 (6)	0.0079 (5)	0.0020 (6)
C17	0.0131 (6)	0.0132 (7)	0.0186 (6)	0.0019 (5)	−0.0024 (5)	−0.0015 (5)
C18	0.0151 (6)	0.0146 (7)	0.0205 (7)	0.0045 (5)	−0.0013 (5)	−0.0051 (5)
C19	0.0146 (6)	0.0155 (7)	0.0153 (6)	0.0000 (5)	−0.0011 (5)	−0.0053 (5)
C20	0.0100 (6)	0.0110 (6)	0.0132 (6)	−0.0010 (5)	0.0006 (5)	0.0001 (5)
C21	0.0106 (6)	0.0111 (6)	0.0136 (6)	−0.0005 (5)	0.0019 (5)	−0.0002 (5)
C22	0.0108 (6)	0.0121 (6)	0.0131 (6)	−0.0005 (5)	0.0006 (5)	0.0018 (5)

### Geometric parameters (Å, °)

**Table:**

Cu1—O2	1.9634 (10)	C6—H6	0.9300
Cu1—O5	1.9548 (10)	C7—H7	0.9300
Cu1—O7^i^	2.3655 (10)	C8—H8A	0.9600
Cu1—O8	1.9667 (10)	C8—H8B	0.9600
Cu1—N1	2.0171 (11)	C8—H8C	0.9600
O1—C1	1.2540 (17)	C9—C10	1.4912 (18)
O2—C1	1.2754 (17)	C10—C15	1.4030 (19)
O3—C8	1.426 (2)	C11—C10	1.3856 (19)
O4—C9	1.2466 (17)	C11—C12	1.3886 (19)
O5—C9	1.2808 (17)	C11—H11	0.9300
O6—C13	1.3634 (17)	C12—C13	1.3876 (19)
O6—C16	1.4355 (17)	C12—H12	0.9300
O7—Cu1^i^	2.3655 (10)	C13—C14	1.399 (2)
O8—H81	0.79 (3)	C14—C15	1.381 (2)
O8—H82	0.83 (2)	C14—H14	0.9300
N1—C17	1.3444 (18)	C15—H15	0.9300
N1—C21	1.3401 (17)	C16—H16A	0.9600
N2—C22	1.3278 (18)	C16—H16B	0.9600
N2—H2A	0.86 (2)	C16—H16C	0.9600
N2—H2B	0.83 (2)	C17—H17	0.9300
C1—C2	1.4985 (19)	C18—C17	1.3833 (19)
C2—C7	1.394 (2)	C18—C19	1.3895 (19)
C3—C2	1.4007 (19)	C18—H18	0.9300
C3—C4	1.383 (2)	C19—H19	0.9300
C3—H3	0.9300	C20—C19	1.3886 (19)
C4—H4	0.9300	C20—C21	1.3903 (18)
C5—O3	1.3650 (17)	C21—H21	0.9300
C5—C4	1.399 (2)	C22—O7	1.2345 (17)
C5—C6	1.391 (2)	C22—C20	1.5053 (18)
C6—C7	1.390 (2)		
			
O2—Cu1—O7^i^	85.44 (4)	H8A—C8—H8B	109.5
O2—Cu1—O8	88.96 (4)	H8A—C8—H8C	109.5
O2—Cu1—N1	93.49 (4)	H8B—C8—H8C	109.5
O5—Cu1—O2	176.73 (4)	O4—C9—O5	123.30 (13)
O5—Cu1—O7^i^	97.41 (4)	O4—C9—C10	119.63 (12)
O5—Cu1—O8	89.07 (4)	O5—C9—C10	117.05 (12)
O5—Cu1—N1	88.22 (4)	C11—C10—C9	120.61 (12)
O8—Cu1—O7^i^	97.34 (4)	C11—C10—C15	118.88 (13)
O8—Cu1—N1	173.84 (5)	C15—C10—C9	120.48 (12)
N1—Cu1—O7^i^	88.50 (4)	C10—C11—C12	121.22 (13)
C1—O2—Cu1	112.27 (9)	C10—C11—H11	119.4
C5—O3—C8	117.64 (13)	C12—C11—H11	119.4
C9—O5—Cu1	114.26 (9)	C11—C12—H12	120.3
C13—O6—C16	116.39 (11)	C13—C12—C11	119.38 (13)
C22—O7—Cu1^i^	134.96 (9)	C13—C12—H12	120.3
Cu1—O8—H82	125.4 (12)	O6—C13—C12	123.72 (13)
Cu1—O8—H81	123.2 (18)	O6—C13—C14	116.04 (12)
H82—O8—H81	103 (2)	C12—C13—C14	120.24 (13)
C17—N1—Cu1	120.44 (9)	C13—C14—H14	120.1
C21—N1—Cu1	120.36 (9)	C15—C14—C13	119.77 (13)
C21—N1—C17	118.32 (12)	C15—C14—H14	120.1
C22—N2—H2A	122.8 (14)	C10—C15—H15	119.7
C22—N2—H2B	120.0 (14)	C14—C15—C10	120.51 (13)
H2B—N2—H2A	116.8 (19)	C14—C15—H15	119.7
O1—C1—O2	123.25 (13)	O6—C16—H16A	109.5
O1—C1—C2	119.15 (13)	O6—C16—H16B	109.5
O2—C1—C2	117.60 (12)	O6—C16—H16C	109.5
C3—C2—C1	121.78 (13)	H16A—C16—H16B	109.5
C7—C2—C1	119.50 (12)	H16A—C16—H16C	109.5
C7—C2—C3	118.72 (13)	H16B—C16—H16C	109.5
C2—C3—H3	119.8	N1—C17—C18	122.43 (12)
C4—C3—C2	120.44 (14)	N1—C17—H17	118.8
C4—C3—H3	119.8	C18—C17—H17	118.8
C3—C4—C5	120.11 (14)	C17—C18—C19	119.00 (13)
C3—C4—H4	119.9	C17—C18—H18	120.5
C5—C4—H4	119.9	C19—C18—H18	120.5
O3—C5—C4	115.72 (14)	C18—C19—H19	120.5
O3—C5—C6	124.15 (14)	C20—C19—C18	118.94 (12)
C6—C5—C4	120.13 (13)	C20—C19—H19	120.5
C5—C6—H6	120.4	C19—C20—C21	118.40 (12)
C7—C6—C5	119.18 (14)	C19—C20—C22	124.05 (12)
C7—C6—H6	120.4	C21—C20—C22	117.45 (12)
C2—C7—H7	119.3	N1—C21—C20	122.83 (13)
C6—C7—C2	121.41 (13)	N1—C21—H21	118.6
C6—C7—H7	119.3	C20—C21—H21	118.6
O3—C8—H8A	109.5	O7—C22—N2	123.72 (13)
O3—C8—H8B	109.5	O7—C22—C20	119.57 (12)
O3—C8—H8C	109.5	N2—C22—C20	116.69 (12)
			
O7^i^—Cu1—O2—C1	−166.69 (9)	C6—C5—O3—C8	−6.9 (2)
O8—Cu1—O2—C1	95.87 (9)	O3—C5—C4—C3	−179.07 (13)
N1—Cu1—O2—C1	−78.48 (9)	C6—C5—C4—C3	0.9 (2)
O7^i^—Cu1—O5—C9	−177.06 (9)	O3—C5—C6—C7	179.92 (13)
O8—Cu1—O5—C9	−79.78 (9)	C4—C5—C6—C7	0.0 (2)
N1—Cu1—O5—C9	94.69 (9)	C5—C6—C7—C2	−0.8 (2)
O2—Cu1—N1—C17	128.61 (11)	O4—C9—C10—C11	−168.45 (13)
O2—Cu1—N1—C21	−62.33 (11)	O4—C9—C10—C15	9.4 (2)
O5—Cu1—N1—C17	−48.59 (11)	O5—C9—C10—C11	9.80 (19)
O5—Cu1—N1—C21	120.47 (11)	O5—C9—C10—C15	−172.30 (12)
O7^i^—Cu1—N1—C17	−146.05 (11)	C9—C10—C15—C14	−178.15 (13)
O7^i^—Cu1—N1—C21	23.01 (10)	C11—C10—C15—C14	−0.2 (2)
Cu1—O2—C1—O1	−0.05 (16)	C12—C11—C10—C9	177.95 (13)
Cu1—O2—C1—C2	179.28 (9)	C12—C11—C10—C15	0.0 (2)
Cu1—O5—C9—O4	8.36 (17)	C10—C11—C12—C13	0.3 (2)
Cu1—O5—C9—C10	−169.82 (9)	C11—C12—C13—O6	179.65 (13)
C16—O6—C13—C12	5.6 (2)	C11—C12—C13—C14	−0.4 (2)
C16—O6—C13—C14	−174.33 (13)	O6—C13—C14—C15	−179.84 (13)
Cu1—N1—C17—C18	168.29 (11)	C12—C13—C14—C15	0.2 (2)
C21—N1—C17—C18	−1.0 (2)	C13—C14—C15—C10	0.1 (2)
Cu1—N1—C21—C20	−166.33 (10)	C19—C18—C17—N1	−1.6 (2)
C17—N1—C21—C20	3.0 (2)	C17—C18—C19—C20	2.2 (2)
O1—C1—C2—C3	178.44 (12)	C21—C20—C19—C18	−0.4 (2)
O1—C1—C2—C7	−2.31 (19)	C22—C20—C19—C18	−176.58 (13)
O2—C1—C2—C3	−0.91 (19)	C19—C20—C21—N1	−2.3 (2)
O2—C1—C2—C7	178.33 (12)	C22—C20—C21—N1	174.18 (12)
C1—C2—C7—C6	−178.58 (12)	O7—C22—C20—C19	170.96 (14)
C3—C2—C7—C6	0.7 (2)	O7—C22—C20—C21	−5.28 (19)
C4—C3—C2—C1	179.42 (13)	N2—C22—O7—Cu1^i^	29.9 (2)
C4—C3—C2—C7	0.2 (2)	N2—C22—C20—C19	−7.8 (2)
C2—C3—C4—C5	−0.9 (2)	N2—C22—C20—C21	175.94 (13)
C4—C5—O3—C8	173.01 (14)	C20—C22—O7—Cu1^i^	−148.77 (10)

Symmetry codes: (i) −*x*+2, −*y*, −*z*+1.

### Hydrogen-bond geometry (Å, °)

**Table:**

*D*—H···*A*	*D*—H	H···*A*	*D*···*A*	*D*—H···*A*
N2—H2A···O1^ii^	0.86 (2)	2.03 (2)	2.8407 (18)	158 (2)
N2—H2B···O4^iii^	0.83 (2)	2.29 (2)	2.9897 (17)	141.4 (18)
O8—H81···O1^iv^	0.79 (3)	1.97 (3)	2.7236 (15)	159 (3)
O8—H82···O4^iv^	0.825 (18)	1.803 (18)	2.6052 (16)	163.9 (18)

Symmetry codes: (ii) −*x*+2, −*y*+1, −*z*+1; (iii) *x*+1/2, −*y*+1/2, *z*+1/2; (iv) −*x*+3/2, *y*−1/2, −*z*+1/2.
